# GC-MS Analysis of Bioactive Compounds Extracted from Plant *Rhazya stricta* Using Various Solvents

**DOI:** 10.3390/plants12040960

**Published:** 2023-02-20

**Authors:** Nabih A. Baeshen, Yaaser Q. Almulaiky, Mohamed Afifi, Ammar Al-Farga, Haytham A. Ali, Naseebh N. Baeshen, Mosleh M. Abomughaid, Aaser M. Abdelazim, Mohammed N. Baeshen

**Affiliations:** 1 Department of Biological Sciences, Faculty of Science, King Abdulaziz University, Jeddah 21589, Saudi Arabia; 2 Department of Chemistry, College of Science and Arts at Khulis, University of Jeddah, Jeddah 21921, Saudi Arabia; 3 Chemistry Department, Faculty of Applied Science, Taiz University, Taiz 3191, Yemen; 4 Department of Biochemistry, Faculty of Veterinary Medicine, Zagazig University, Zagazig 44519, Egypt; 5 Department of Biochemistry, College of Science, University of Jeddah, Jeddah 21959, Saudi Arabia; 6 Department of Biology, College of Sciences and Arts at Khulais, University of Jeddah, Jeddah 21959, Saudi Arabia; 7 Laboratory Medical Sciences, College of Applied Medical Sciences, University of Bisha, Bisha 61922, Saudi Arabia; 8 Department of Biology, College of Science, University of Jeddah, Jeddah 21959, Saudi Arabia

**Keywords:** phytochemical compounds, different solvents, *Rhazya stricta*, alkaloid, plant

## Abstract

Worldwide, human beings have traditionally employed many folkloric herbal resources as complementary and alternative remedies, and these remedies have played a pivotal role in modern medicines for many decades, as scientists have used them to develop drugs. We studied the effects of employing solvents with varying polarity on the yields of phytochemical components extracted from the plant *Rhazya stricta*. We used chloroform–methanol (1:1), methanol, ethanol, diethyl ether, and ethyl acetate as extraction solvents. The results showed that the efficiencies of the solvents at extracting phytochemical compounds were in this order: chloroform–methanol < ethanol < methanol < diethyl ether < ethyl acetate extract. The chloroform–methanol extract produced the highest concentration of phenolic and flavonoid contents among the five solvents tested (13.3 mg GAE/g DM and 5.43 CE/g DM). The yields of the extracted phytochemical compounds ranged from 47.55 to 6.05%. The results revealed that the properties of the extraction solvents considerably impacted the extraction yield and the phytochemical components of the *R. stricta* extract. Furthermore, compared with the other solvents, the chloroform–methanol extraction led to the highest yield (47.55%) and to more phytochemical substances being extracted. The aim of this study is to investigate the phytochemical compounds extracted from *R. stricta* with different solvents that have different polarities.

## 1. Introduction

People have used natural medicinal plants as self-medication to treat diseases for many decades; however, scholars have debated the biologically-active molecules, plant-derived molecules, and mechanisms of action occurring in natural medicines for years. It is believed that people commonly employ folkloric herbal remedies as a source of innovative medications in folk medicine, and they have used these remedies, which have shown promising potential, to treat various human and animal diseases [1,2]. On the Arabian Peninsula, Saudi Arabian plants have a rich biological diversity and represent a significant genetic resource for both agriculture and medicinal plants. Due to its geographic location and characteristically dry weather, a large number of these plants grow under adverse weather conditions, meaning that their genomes are remarkably unique; thus, individuals use them to treat various conditions [3,4].

Primary metabolites are found in all plants, while secondary metabolites help a particular plant species interact with its environment. Plant-specific and genetically determined, the contents of physiologically active substances are additionally influenced by cultivation practices, diseases and pests, climate, developmental stage, ecology, and the time of day that the material is gathered [5]. Saudi Arabia’s harsh environmental conditions have forced plants to evolve coping mechanisms. However, according to phytochemistry, this causes high quantities of secondary metabolites such as polyphenols, flavonoids, tannins, terpenes, alkaloids, and saponins and their glycosides [6].

Current pharmacology explains the importance of natural products for developing novel drugs. Many natural compounds have been utilized as the foundation for the creation of medications and are still in use today to treat various diseases. However, the use of modern drugs entails a multitude of challenges, including severe side effects and drug resistance to antibiotics or even anti-cancer medications, which requires the development of novel medications. For instance, typical NSAIDs are well-known for their side effects, which include gastrointestinal hemorrhage and cardiovascular events [7]. Therefore, it is necessary to develop new NSAIDs with fewer side effects. In addition to antibiotic side effects, unchecked use increases the chance that bacteria will evolve resistance, which raises the risk of fatal infections [8]. In Saudi Arabia, cancer incidence has increased in recent years; breast, uterine, bladder, and colon cancer rates have risen roughly 10 times. Thyroid cancer incidence has increased by a factor of 26. From 5% in 1990 to 12% in 2016 [9], Saudi Arabia had an increase in cancer-related fatalities. An analysis of the ethnopharmacology of Saudi Arabian plants revealed that Saudi residents depend on conventional and contemporary therapies [10]. However, there are no data on the phytochemical components derived from *Rhazya stricta* in SA, despite the fact that various articles discuss traditional medicines in Saudi Arabia [11,12]. Therefore, it is possible to discover innovative hits for medication development by fusing conventional wisdom with contemporary pharmacognostic research, leading to the evidence-based application of traditional medicines and novel drug development. 

*Rhazya stricta* is a classic shrub that is toxic, low, erect, and glabrous. It is one of the most common medicinal shrubs in the desert of the Arab Peninsula, including Saudi Arabia, and is used in herbal medicines to treat various diseases [13]. Recently, scientists have used its extracted materials in the formulation of silver nanoparticles, which have a role in fighting mosquito vectors and multiple pathogens [14]. *R. stricta* contains glycosides, alkaloids, tannins, and triterpenes, which are considered to be a rich source of indole alkaloids [15,16]. Indole alkaloid compounds generally exhibit antinociceptive, antitumor, anti-inflammatory, antioxidant, and antimicrobial antihypertensive properties [17]. Scientists have identified more than 100 alkaloids from *R. stricta* using phytochemical analysis methods [18]. Based on these aforementioned facts, we aimed to investigate the phytochemical compounds that are extracted from *R. stricta* with different solvents (methanol–chloroform (1:1), diethyl ester, methanol, ethanol, and ethyl acetate) and the identification of bioactive compounds. Using multiple solvents to extract compounds from *R. stricta* will provide us with opportunities to discover various bioactive compounds with therapeutic potential. 

## 2. Results and Discussion

### 2.1. Phenolic and Flavonoids Contents

Plant potential antioxidant activity is proportional to the amount of cell-reinforcing chemicals present, such as phenolic compounds that are capable of catalyzing free radical scavenging [19]. To extract phenolic and flavonoid chemicals, the appropriate solvent must be utilized. Table 1 shows the capacity of several solvents to extract phenolic and flavonoid compounds from *R. stricta*. We tested methanol, ethanol, ethyl acetate, diethyl ether, and chloroform–methanol (1:1) to determine the best solvent to extract phenolic and flavonoid chemicals. Chloroform–methanol produced the highest concentration of phenolic compounds among the five solvents tested (13.3 mg GAE/g DM), and it produced a higher flavonoid content concentration (5.43 CE/g DM). Chloroform–methanol was the best solvent for extracting polyphenolic chemicals from the plants due to its ability to inhibit polyphenol oxidase activity. This enzyme is responsible for polyphenols’ oxidation and dispersion efficiency [20]. In Caesalpinia decapetala [21], Portulacaceae [19], and Morus nigra and Artocarpus heterophyllus leaves [22], scientists have used methanol (70%) extracts to investigate antioxidant properties and flavonoid components. We performed a correlation study on the phenolic and flavonoid content of *R. stricta* extracts. It was revealed that there was a 0.995 connection between the phenolic and flavonoid contents, suggesting that, in *R. stricta*, flavonoids are the predominant phenolic group. The results are comparable to the extraction of phenolics from Pisang Mas, Guava, and Limnophila aromatica [23,24].

### 2.2. Extraction with Ethanol Solvent and Identification of Compounds Using GC/MS

**Figure 1:**
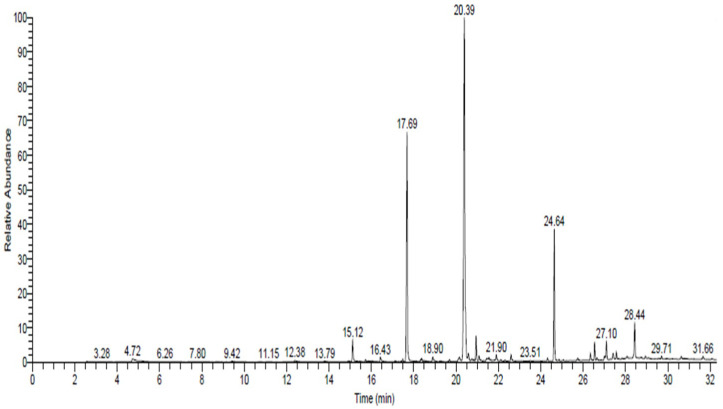
GC-MS chromatograms of *Rhazya stricta* extracted with ethanol solvent.

Table 2 and Figure 1 show 18 compounds found in *R. stricta* extract using an ethanol solvent. We used the peak area percentage to indicate the relative concentration of each compound. The main compounds identified based on the relative contents were Methyl octadeca-17-enoate (46.32%), Methyl hexadecanoate (Methyl palmitate) (24.22%), (-)-1,2-Didehydroaspidospermidine (11.39%), and Strictamine (3.44%). Most of the compounds extracted with ethanol were unsaturated fatty acids. Methyl hexadecanoate plays a vital role in modulating anti-inflammatory responses in macrophages [25]. Additionally, it affects human semen quality [26]. Further, 1,2-Didehydroaspidospermidine is a bioactive alkaloid extracted from many plants, and scientists have used it as a target for synthesis [27]. Finally, Strictamine has promising and significant antibacterial potential against *Acinetobacter baumannii* [28]. Our results are in accordance with previous reports showing the fatty acid profile of *R. stricta* [16]. These results suggest a positive biological effect of the bioactive materials extracted from *R. stricta* with an ethanol solvent. Similarly, the high fatty acid content extracted from *R. stricta* demonstrates its volatile flavors, which scholars have previously detected [29].

### 2.3. Extraction with Methanol Solvent and Identification of Compounds Using GC/MS

**Figure 2:**
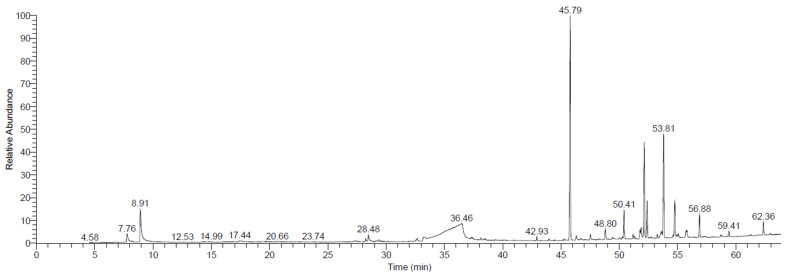
GC-MS chromatograms of *Rhazya stricta* extracted with methanol solvent.

Table 3 and Figure 2 present the 18 compounds extracted from R. stricta with the methanol solvent. The main compounds identified based on relative contents were (-)-1,2-Didehydroaspidospermidine (28.37%), Methyl aspidospermidine-3-carboxylate (14.27%), quebrachamine (11.96%), and 3-Ethylpiperidine (5.63%). Most of the compounds extracted with methanol were alkaloids; similarly, previous data showed the existence of alkaloids in R. stricta [15,30]. Additionally, genetic diversity can affect the plant content of alkaloids [31]. Alkaloids are a rich source of the materials used for drug discovery and formulation; thus, scientists have examined various alkaloids for their anticancer and antiproliferative activities [32,33]. The results of another study elucidated their role in providing protection to animals subjected to UV radiation [34]. The results obtained in the present study emphasize the potential therapeutic use of R. stricta, especially as a potent source of alkaloids, and the potential for researchers to discover multiple bioactive materials with therapeutic properties against different malignancies.

### 2.4. Extraction with Diethyl Ether Solvent and Identification of Compounds Using GC/MS

**Figure 3:**
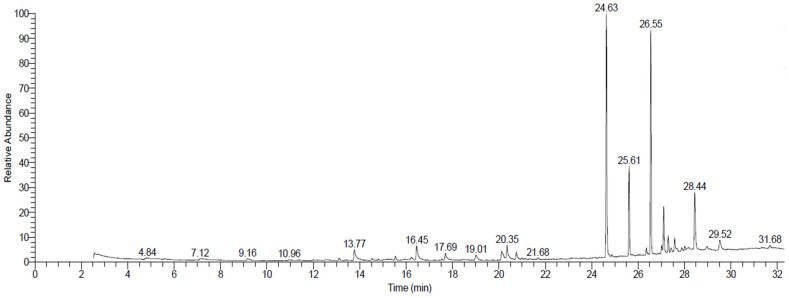
GC-MS chromatograms of *Rhazya stricta* extracted with diethyl ether solvent.

Table 4 and Figure 3 show the 15 compounds found in *R. stricta* extract using the diethyl ether solvent. The main compounds identified based on the relative contents were (-)-1,2-Didehydroaspidospermidine (26.76%), squalene (22.49%), Di-n-2-propylpentylphthalate (9.19%), and quebrachamine (5.49%). Most of the compounds extracted with diethyl ether were alkaloids and triterpenes. Scientists have shown that triterpenes exist in *R. stricta* via cheminformatics studies that they performed to determine the bioactive molecules responsible for their therapeutic potential [35]. Scholars have revealed that triterpenes have various medicinal uses due to their antitumor activities [36], inhibitory effect on nitric oxide (NO) production [37], anti-inflammatory activities [38], and antineoplastic activities [39]. Although *R. stricta* has high therapeutic potential, its phthalic acid content has provoked discussions about the adverse effect of this bioactive compound [40,41].

Moreover, scientists have detected a high amount of squalene in *R. stricta*. Squalene is a polyunsaturated hydrocarbon with multiple bioactivities, including skin hydration, acting as an emollient agent and drug carrier, and having antioxidant and detoxification properties [42]. Recently, scholars discovered the important role of squalene as an adjuvant for influenza vaccines [43], and they determined its role in the treatment of cardiovascular disease through its statin-like action [44]. Quebrachamine, another indole alkaloid extracted from *R. stricta*, has blocking activities against the adrenergic nerves of urogenital tissues [45]. Our results are in accordance with previous reports that also detected quebrachamine in *R. stricta* [16]. The bioactive materials extracted from *R. stricta* with diethyl ester tended to have important activities for therapeutic uses; Sultana and Khalid, 2010, reported the same prospect [46]. All the previously-mentioned records emphasize the therapeutic potential of *R. stricta* regarding its isolated and extracted bioactive compounds.

### 2.5. Extraction with Chloroform–Methanol Solvent and Identification of Compounds Using GC/MS

**Figure 4:**
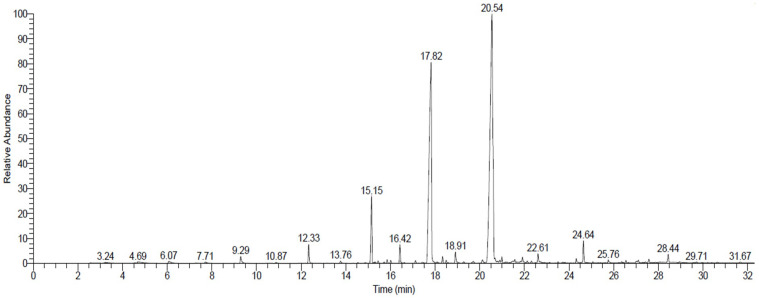
GC-MS chromatograms of *Rhazya stricta* extracted with chloroform–methanol solvent.

Table 5 and Figure 4 show the 10 compounds that we found in *R. stricta* via extraction with the chloroform–methanol solvent. The compounds identified based on the relative contents were methyl stearate (47.55%), Methyl palmitate (35.23%), methyl tetradecanoate (6.03%), (-)-1,2-Didehydroaspidospermidine (1.53%), and Methyl laurate (1.46%). Most of the compounds extracted with chloroform–methanol were fatty acids and alkaloids. Our study’s results are comparable to those of previous studies, whereby the authors extracted more than 100 alkaloid compounds from *R. stricta* [47]. We found that methyl stearate, the fatty acid that we extracted most often from *R. stricta* with chloroform–methanol, had a regulatory effect on the calcium-activated chloride channels, which has sparked debate on its use in drug synthesis and fabrication [48]. Additionally, it has anti-inflammatory activities through its ability to downregulate the proinflammatory response [49]. Moreover, methyl stearate has several uses in biological and medical research [50]. Another bioactive compound, methyl tetradecanoate, a fatty acid extracted from *R. stricta*, has contraceptive activities [51]. The previously-mentioned citations confirm the potential of the extracted *R. stricta* bioactive compounds to be a potent therapeutic compound.

### 2.6. Extraction with Ethyl Acetate Solvent and Identification of Compounds Using GC/MS

**Figure 5:**
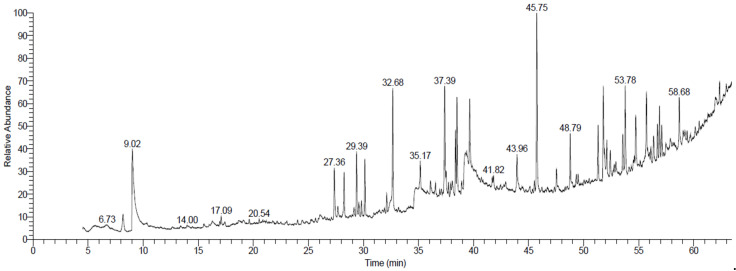
GC-MS chromatograms of *Rhazya stricta* extracted with ethyl acetate solvent.

Table 6 and Figure 5 show the 10 compounds extracted from *R. stricta* using the ethyl acetate solvent. The main compounds identified based on the relative contents were (-)-1,2-Didehydroaspidospermidine (6.05%), 3-ethylpyridine (4.01%), N-ethyl-desoxy-veratramine (3.11%), aR-Turmerone (2.10%), oleic acid (2.16%), and vitamin E (1.94%). The *R. stricta* extraction with the ethyl acetate solvent resulted in a higher oleic acid content. The results are comparable to those of previous studies that showed the existence of oleic acid in *R. stricta* [52]. As an omega-9 unsaturated fatty acid, oleic acid regulates female fertility and is involved in germ cell growth and development. It contributes to oocyte preimplementation and embryo growth [53].

Moreover, it plays a beneficial role in diminishing the incidence of cardiovascular disorders, carcinogenesis, liver dysfunctions, and intestinal inflammations [54]. Additionally, it has a potent ability to mitigate inflammatory responses in sepsis, has antioxidant power, takes antiparasitic action against *Acanthamoeba castellanii* trophozoites, and promotes the differentiation of neural cells in human endometrial stem cells [55,56]. Furthermore, oleic acid ameliorates induced hepatocellular lipotoxicity [57], acts as a carrier for anticancer drugs [58], upregulates myosin heavy chain-1 expression, and elevates the mitochondrial mass in myoblasts [59]. Its high oleic acid content makes *R. stricta* a possible medicinal plant for many diseases. Also, we extracted vitamin E from *R. stricta*; the biological activities and the importance of vitamin E are well known, and researcher studies have recently and extensively shown its antioxidant power [60,61]. Recently, scholars have found that lower serum levels of α-tocopherol and lycopene are more associated with high pain and disability in osteoarthritis patients than in healthy controls [62]. Moreover, its administration after surgical operations enhances the osseointegration of stainless-steel implants in vivo [63]. The obtained results show that *R. stricta* is a potent source of vitamin E and, thus, can be a powerful source of antioxidants.

### 2.7. Comparison between Extraction Percentage of the Phytochemical Compounds Using Different Solvents

The results shown in Table 7 indicate that the main bioactive compounds extracted by different solvents belong to families of alkaloids, fatty acids, triterpene, antimicrobials, vitamin E, and antibiotics. These bioactive compounds could open new horizons to more in-depth studies to evaluate the mode of action of the compounds that are necessary to pave the way for clinical trials. The isolation and purification of these compounds are needed to assess their mode of action with in vitro studies to better understand their activities. The discrepancies in the RT that are obvious for bioactive compounds extracted using different solvents could be attributed to variances in the polarity of various plant chemicals, as described by Jayaprakasha et al. [64]. As a result of this variation, the solubility of the solvent that was employed differed, and the RT of the bioactive compounds which were extracted varied depending on the kind of solvent used [65]. These results agree with Swamy et al. [66], who used different solvents (methanol, acetone, and hexane) to extract *Plectranthus amboinicus* leaves. They revealed that the retention time of the same compound might vary in the same column under the same analytical conditions with a different solvent. For instance, tetrapentacontane appears in the methanol extract at Rt 72.63 min and in the hexane extract at Rt 92.76 min. Pentaconsane appears in the ethanol extract at Rt 75.78 min and in the hexane extract at Rt 81.95 min. Squalane appears in the methanol extract at Rt 86.54 min and in the hexane extract at Rt 75.43 min [66].

## 3. Materials and Methods

### 3.1. Collection of Plant Samples and Preparation

We collected *R. stricta* plant materials from the Ghola area at Osfan with the coordinates N: 21.935.1966 and E: 39.305869. We brought the collected samples to the laboratory, separated the leaves from the stems, washed them with running tap water, and left them to dry in the shade at the laboratory for three days. When the leaves were completely dehydrated, we placed them in a blender, ground them to a fine powder, and kept them at room temperature for further use.

### 3.2. Sample Extraction

We extracted 100 g of fine powder using 500 mL of absolute ethanol, methanol, diethyl ether, a chloroform–methanol mixture (1:1, *v*/*v*), or ethyl acetate. We ultrasonicated all the samples in a water bath at 40 °C for three hours, soaked them in a shaking water bath at 70 °C for 24 h until the solvent became colorless, filtered them through Whatman filter paper No.2, and analyzed them with GC-MS.

### 3.3. Total Phenolic Content

We used the method explained by [67] to determine the total phenolic content of the plant. Firstly, we introduced 100 μL of the Folin–Ciocalteu reagent to 100 μL of the plant extract and 800 μL of distilled water, and left the solution for 5 min at room temperature. We then added 500 μL of sodium carbonate (15%, *w*/*v*) to the reaction mixture. Finally, we measured the absorbance at 750 nm after 30 min. The results are represented in mg gallic acid equivalent per gram of dry matter (mg GAE/g DM).

### 3.4. Total Flavonoid Content

We used the method described by [68] to determine the flavonoid content. Firstly, we combined 250 μL of plant extract, 1.25 μL of distilled water, and 75 μL of NaNO_2_ solution (5%, *w*/*v*) in a reaction mixture and allowed it to stand for 6 min. Then, we added 150 μL of an AlCl_3_ solution (10%, *w*/*v*), 0.5 mL of 1 M NaOH, and 275 μL of distilled water to the reaction mixture and allowed it to stand for 5 min. Finally, we recorded the absorbance at 510 nm. Then, we calculated the results as mg catechin equivalent/g dry matter (mg CE/g DM) and used a catechin solution as the standard.

### 3.5. Gas Chromatography-Mass Spectrometry (GC-MS) Analysis

We determined the chemical compositions of the samples using a Thermo Scientific Trace GC1310-ISQ mass spectrometer with a direct capillary column TG–5MS (30 m × 0.25 mm × 0.25 m film thickness). Initially, we maintained the column oven at 50 °C; then, we increased the temperature by 5 °C/min to 230 °C, which we held for 2 min, and then by 30 °C/min to 290 °C, which we also maintained for 2 min. Next, we held the temperature of the injector and MS transfer lines at 250 and 260 °C, respectively. We used helium as a carrier gas at a constant flow rate of 1 mL/min. The solvent delay was 3 min, and we automatically injected 1 μL of the diluted samples using Autosampler AS1300 coupled with GC in the split mode. We collected EI mass spectra at 70 eV ionization voltages over the range of *m*/*z* 40–1000 in full scan mode. Next, we set the ion source temperature to 200 °C. Finally, we identified the components by comparing the components’ retention times and mass spectra to those of the WILEY 09 and NIST 11 mass spectral databases.

## 4. Conclusions

This study investigated the effects of solvents with different polarities on the phytochemical compounds derived from *R. stricta*. The solvents that were used included chloroform–methanol, ethanol, methanol, diethyl ether, and ethyl acetate. The results revealed that chloroform–methanol use resulted in a high extraction yield of extracted phytochemical compounds (13.3 ± 0.86 mg/g phenolic content and 5.43 ± 0.89 mg/g flavonoid content). The majority of the compounds extracted with chloroform–methanol were Methyl stearate (47.55%), which plays a regulatory role in the calcium-activated chloride channels and has anti-inflammatory activities through its ability to downregulate the proinflammatory response, and hexadecanoic acid (35.23%), which has a vital role in modulating anti-inflammatory reactions in macrophages and affects human semen quality. Therefore, the properties of the extraction solvents play an important role in determining the effectiveness of phytochemical compound extraction. Furthermore, the extracted bioactive compounds revealed the medicinal potential of *R. stricta* for female reproduction disorders, cardiovascular disease, obesity, inflammatory conditions, and hepatic disorders. Moreover, it is a rich source of antioxidants, alkaloids, and beneficial unsaturated fatty acids. Therefore, it is possible to separate, isolate, and characterize all of the phytocomponents found in *R. stricta* in order to identify novel drugs and study their therapeutic benefits. Future studies will concentrate on separating and characterizing particular compounds from *R. stricta* crude extracts and testing them in living organisms to better understand their activities.

## Figures and Tables

**Table 1:** Total phenolics and flavonoids of *Rhazya stricta* extracted with different solvents. Values are the means of three replicates ± SD.

Solvent	Phenolic Content (mg/g)	Flavonoid Content (mg/g)
Chloroform–methanol	13.3 ± 0.86	5.43 ± 0.89
Methanol	6.4 ± 0.24	2.75 ± 0.43
Diethyl ether	2.5 ± 0.16	1.12 ± 0.52
Ethyl acetate	1.61 ± 0.09	0.63 ± 0.39
Ethanol	8.32 ± 0.45	3.87 ± 0.21

**Table 2:** Phytochemical compounds of *Rhazya stricta* extracted with ethanol solvent.

	Identified Name	Rt* (min)	Peak Area (%)
1	Methyl tetradecanoate	15.12	2.16
2	Methyl pentadecanoate	16.43	0.60
3	Methyl palmitate	17.69	24.22
4	Methyl 15-methylhexadecanoate	18.90	0.68
5	(Z)-1,1-dimethoxyoctadec-9-ene	20.16	0.68
6	Methyl octadeca-17-enoate	20.39	46.32
7	Methyl linoleate	20.59	0.52
8	Methyl 9,12,15-octadecatrienoate	20.95	2.22
9	Ethyl octadec-9-enoate	21.09	0.65
10	Methyl 10-trans,12-cis-octadecadienoate	21.90	0.79
11	Methyl 18-methylnonadecanoate	22.60	0.79
12	(-)-1,2-Didehydroaspidospermidine	24.64	11.39
13	2,4,4-Trimethylcyclopenten-3-one	26.35	0.61
14	Squalene	26.54	1.47
15	8,9,10,11-Tetrahdro-7-methylbenz[c]acridine	27.01	0.40
16	Quebrachamine	27.1	1.69
17	2á,3à-Dihydrovincadifformine	27.57	0.68
18	Strictamine	28.44	3.44

Rt*: the retention time (RT) of a single compound. The time it takes for the compound to go through the column is affected by its length, temperature, and the carrier gas’s flow rate.

**Table 3:** Phytochemical compounds of *Rhazya stricta* extracted with methanol solvent.

	Identified Name	Rt* (min)	Peak Area (%)
1	N,N-Dimethyl-1-cyclohexen-1-amine	7.76	1.54
2	3-Ethylpiperidine	8.92	5.63
3	2,6-Dimethyl-3-(methoxymethyl)-p-benzoquinone	28.48	0.99
4	1,3,4,5-Terahydroxy-cyclohexanecarboxylic acid	33.22	1.03
5	Mome Inositol	36.46	5.26
6	Halofantrine	42.93	0.51
7	(-)-1,2-Didehydroaspidospermidine	45.79	28.37
8	2-Ethyl-3-[2′-3″-Ethylpiperiduethyl]Indole	48.80	1.41
9	3-cyano-5,5-dimethyltetrafura N-2-one	50.41	3.47
10	Eburnamenine	51.77	1.02
11	8,9,10,11-Tetrahydro-7-methylbenz[c]acridine	51.87	1.44
12	Quebrachamine	52.14	11.96
13	Clindamycin	52.39	4.43
14	2-ethyl-3-[2′-3″-ethyl piperidu ethyl] indole	53.64	1.70
15	Methyl aspidospermidine-3-carboxylate	53.81	14.27
16	2-Amino-4-(4-ethoxy-phenyl)-6-methoxy-pyridine-3,5-dicarbonitrile	54.76	5.04
17	Strictamine	55.79	1.72
18	1-Oxa-2-azaspiro[5.5]undecane-3-carbonitrile,2-cyclohexyl-4-(trimethylsilyloxymethyl)-	62.36	1.75

**Table 4:** Phytochemical compounds of *Rhazya stricta* extracted with diethyl ether solvent.

	Identified Name	Rt* (min)	Peak Area (%)
1	Hexadecanal	16.45	2.62
2	Methyl palmitate	17.69	1.37
3	Olealdehyde	19.01	1.20
4	Methyl octadeca-17-enoate	20.35	2.07
5	1-O-butyl 2-O-heptan-3-yl benzene-1,2-dicarboxylate	20.75	1.21
6	(-)-1,2-Didehydroaspidospermidine	24.63	26.76
7	Di-n-2-propylpentylphthalate	25.61	9.19
8	Aspidospermidine	26.35	0.92
9	Squalene	26.55	22.49
10	Quebrachamine	27.10	5.49
11	Dotriacontane	27.30	1.91
12	Methyl 2,3-didehydroaspidospermidine-3-carboxylate	27.58	2.15
13	Yohimban-17-one	28.97	0.77
14	Vitamin E	29.52	2.16
15	Hexaphenylcyclotrisiloxane	31.68	0.57

**Table 5:** Phytochemical compounds of *Rhazya stricta* extracted with chloroform–methanol solvent.

	Identified Name	Rt* (min)	Peak Area (%)
1	Decanoic acid, methyl ester	9.29	0.71
2	Methyl laurate	12.33	1.46
3	Methyl tetradecanoate	15.15	6.03
4	Methyl 12-methyltetradecanoate	16.42	1.43
5	Methyl palmitate	17.82	35.23
6	Methyl stearate	20.54	47.55
7	Methyl arachisate	22.61	0.76
8	(-)-1,2-Didehydroaspidospermidine	24.64	1.53
9	Methyl lignocerate	25.76	0.26
10	Strictamine	28.44	0.66

**Table 6:** Phytochemical compounds of *Rhazya stricta* extracted with ethyl acetate solvent.

	Identified Name	Rt* (min)	Peak Area (%)
1	3-Ethylpyridine	9.03	4.01
2	2(4H)-Benzofuranone,5,6,7,7a-tetrahydro-4,4,7a-trimethyl-, (R)-	27.36	1.88
3	Neophytadiene	28.25	1.55
4	aR-Turmerone	29.39	2.10
5	Hexahydrofarnesyl acetone	30.14	1.79
6	Oleic Acid	39.22	2.16
7	(-)-1,2-Didehydroaspidospermidine	45.75	6.05
8	N-Ethyl-desoxy-veratramine	53.78	3.11
9	Aspidofractinin-3-one	54.74	2.04
10	Vitamin E	58.69	1.94

**Table 7:** Comparison of phytochemical compounds of *Rhazya stricta* extracted with various solvents.

Kind	Bioactive Compounds	Ethanol		Methanol		Diethyl Ether		Chloroform–Methanol		Ethyl Acetate Extract	
		R T	Area (%)	R T	Area (%)	R T	Area (%)	R T	Area (%)	R T	Area (%)
	N,N-Dimethyl-1-cyclohexen-1-amine	-	-	7.76	1.54	-	-	-	-	-	-
	3-Ethylpiperidine	-	-	8.92	5.63	-	-	-	-	-	-
	Quebrachamine	27.1	1.69	52.14	11.96	27.1	5.49	-	-	-	-
	Clindamycin	-	-	52.39	4.43	-	-	-	-	-	-
**Alkaloids**	(-)-1,2-Didehydroaspidospermidine	24.64	11.39	45.79	28.37	24.63	26.76	24.64	1.53	45.75	6.05
	Aspidospermidine	-	-	-	-	26.3	0.92	-	-	-	-
	Yohimban-17-one	-	-	-	-	28.97	0.77	-	-	-	-
	Strictamine	28.44	3.44	55.79	1.72	-	-	28.88	0.66	-	-
	Methyl tetradecanoate	15.12	2.16	-	-	-	-	15.15	6.03	-	-
	Methyl pentadecanoate	16.43	0.60	-	-	-	-	-	-	-	-
	Methyl palmitate	17.69	24.22	-	-	17.69	1.37	17.82	35.23	-	-
	Methyl octadeca-17-enoate	20.39	46.32	-	-	20.35	2.07	-	-	-	-
**Fatty acid**	Methyl linoleate	20.59	0.52	-	-	-	-	-	-	-	-
	Methyl 9,12,15-octadecatrienoate	20.95	2.22	-	-	-	-	-	-	-	-
**Triterpene**	Squalene	26.54	1.47	-	-	26.55	22.49	-	-	-	-
**Antimicrobial**	1-O-butyl 2-O-heptan-3-yl benzene-1,2-dicarboxylate	-	-	-	-	20.75	1.21	-	-	-	-
	Di-n-2-propylpentylphthalate	-	-	-	-	25.6	9.19	-	-	-	-
**Vitamin E**	Vitamin E	-	-	-	-	29.52	2.16	-	-	58.69	1.94
**Antibiotic**	Clindamycin	-	-	52.39	4.43	-	-	-	-		

## Data Availability

Data is contained within the article.

## Abbreviations

GAE: gallic acid equivalent; DM: dry matter; CE: catechin.
